# Experimental Models of Short Courses of Liposomal Amphotericin B for Induction Therapy for Cryptococcal Meningitis

**DOI:** 10.1128/AAC.00090-17

**Published:** 2017-05-24

**Authors:** Jodi Lestner, Laura McEntee, Adam Johnson, Joanne Livermore, Sarah Whalley, Julie Schwartz, John R. Perfect, Thomas Harrison, William Hope

**Affiliations:** a Antimicrobial Pharmacodynamics and Therapeutics, Department of Molecular and Clinical Pharmacology, University of Liverpool, Liverpool, United Kingdom; b Charles River Laboratories, Davis, California, USA; c Department of Medicine, Duke University, Durham, North Carolina, USA; d Research Centre for Infection and Immunity, St. George's University of London, London, United Kingdom

**Keywords:** liposomal amphotericin B, pharmacokinetics, pharmacodynamics, Cryptococcus neoformans, cryptococcal meningitis, meningoencephalitis

## Abstract

Cryptococcal meningoencephalitis is a rapidly lethal infection in immunocompromised patients. Induction regimens are usually administered for 2 weeks. The shortest effective period of induction therapy with liposomal amphotericin B (LAMB) is unknown. The pharmacodynamics of LAMB were studied in murine and rabbit models of cryptococcal meningoencephalitis. The concentrations of LAMB in the plasma and brains of mice were measured using high-performance liquid chromatography (HPLC). Histopathological changes were determined. The penetration of LAMB into the brain was determined by immunohistochemistry using an antibody directed to amphotericin B. A dose-dependent decline in fungal burden was observed in the brains of mice, with near-maximal efficacy achieved with LAMB at 10 to 20 mg/kg/day. The terminal elimination half-life in the brain was 133 h. The pharmacodynamics of a single dose of 20 mg/kg was the same as that of 20 mg/kg/day administered for 2 weeks. Changes in quantitative counts were reflected by histopathological changes in the brain. Three doses of LAMB at 5 mg/kg/day in rabbits were required to achieve fungicidal activity in cerebrospinal fluid (cumulative area under the concentration-time curve, 2,500 mg · h/liter). Amphotericin B was visible in the intra- and perivascular spaces, the leptomeninges, and the choroid plexus. The prolonged mean residence time of amphotericin B in the brain suggests that abbreviated induction regimens of LAMB are possible for cryptococcal meningoencephalitis.

## INTRODUCTION

Cryptococcal meningitis is a common and frequently lethal disease in patients with HIV/AIDS (1). Rapid fungicidal activity in cerebrospinal fluid (CSF) is associated with better clinical outcomes and improved survival (2). Amphotericin B deoxycholate (DAMB) is the most potent amphotericin B formulation on a milligram-milligram basis (3, 4). While effective, DAMB is toxic and associated with significant infusion-related toxicity, nephrotoxicity, and anemia (5, 6). Furthermore, DAMB is not orally bioavailable and must be injected. The need for rapid, reliable monitoring for side effects and for intravenous administration means that amphotericin B-based treatment is simply not possible in many resource-poor settings. Hence, the best current therapy cannot be administered to patients in many countries where the prevalence of cryptococcal meningitis is the highest. In these cases, the only alternative agent is fluconazole, but even with the use of high doses (800 to 1,200 mg/day), the fungicidal activity in CSF and clinical outcomes are suboptimal (7, 8). Alternative approaches are urgently required.

There is surprisingly little evidence for the use of liposomal amphotericin B (LAMB) for cryptococcal meningitis. Preclinical and clinical data suggest that 3 to 6 mg/kg of body weight/day is a safe and effective regimen (9, 10). Typically, the duration of amphotericin B-based induction regimens is 2 weeks, based primarily on surrogate mycological markers of early fungicidal activity such as CSF sterilization (11, 12). The shortest duration of LAMB that is maximally effective is not known. We recently demonstrated that an abbreviated course of DAMB (3 days) may be as effective as 2 weeks of therapy (13) and that short courses of DAMB (in combination with fluconazole) are associated with rapid clearance of the CSF in patients with cryptococcal meningitis (14). Thus, there are a precedent and a rationale for examining the safety and efficacy of abbreviated regimens of LAMB as induction therapy for cryptococcal meningitis.

Here, we used two previously described (15, 16) and well-characterized laboratory animal models of cryptococcal meningitis to study the pharmacodynamics of abbreviated courses of liposomal amphotericin B. Our principal goal was to provide the experimental evidence underpinning phase II and III clinical trials examining the efficacy of abbreviated regimens of LAMB.

## RESULTS

### Dose-exposure-response relationships in mice.

**FIG 1:**
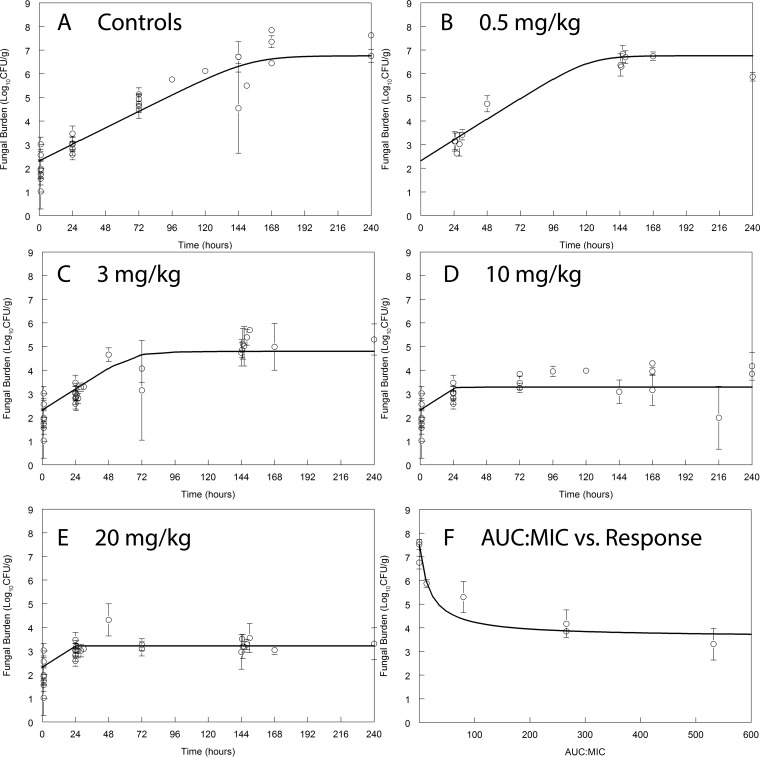
Pharmacodynamics of LAMB in cohorts of mice receiving 0.5 (B), 3 (C), 10 (D), and 20 (E) mg/kg/day i.v. The AUC/MIC ratio at steady state versus the observed fungal density at the end of the experiment (time = 240 h) is shown in panel F. All data are means ± standard deviations of results from groups of three mice.

Liposomal amphotericin B was well tolerated in mice with no observed toxicity following rapid intravenous (i.v.) injection. There was a clear dose-response relationship with doses of 0.5 to 20 mg/kg/day. Fungicidal activity was not observed (i.e., we did not observe a decline in the number of log_10_ CFU/g following daily therapy). Rather, a fungistatic effect was seen whereby the infection at the time of drug administration 24 h postinoculation was stabilized. Near-maximal antifungal activity was observed following treatment with 10 to 20 mg/kg/day and with an AUC/MIC ratio of approximately 100 (Fig. 1).

**FIG 2:**
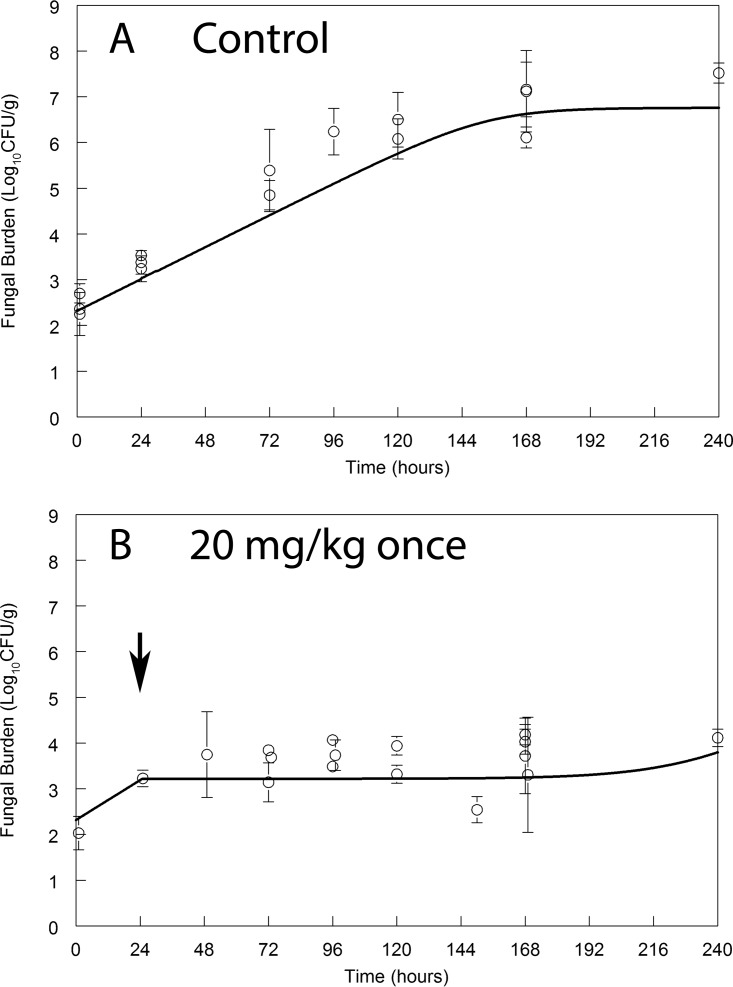
Pharmacodynamics of vehicle control (A) and LAMB (B) following the administration of a single dose of 20 mg/kg i.v. to mice with cryptococcal meningoencephalitis. Data are the means ± standard deviations of results from groups of three mice. The solid line represents the fit of the mathematical PK-PD model (parameters summarized in Table 1). The black arrow denotes the time of drug administration relative to inoculation (which occurred at time zero).

**FIG 3:**
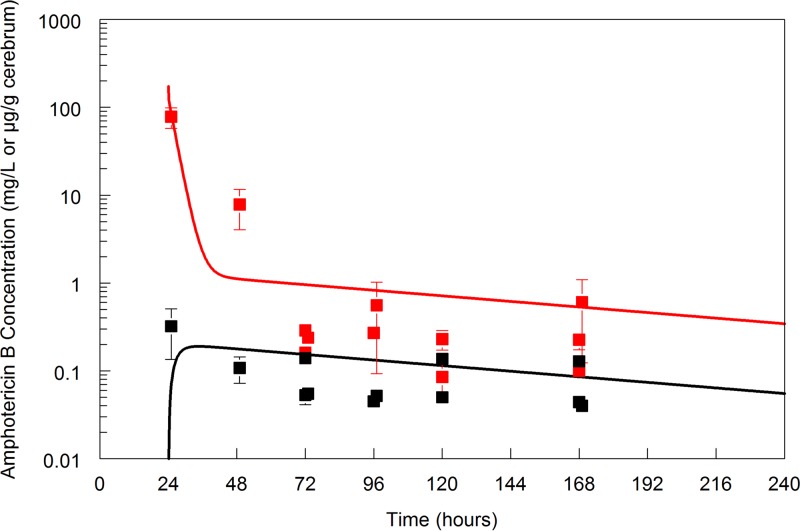
Pharmacokinetics of LAMB in murine plasma (red line and red data points) and cerebrum (black line and black data points) in cohorts of mice infected with Cryptococcus neoformans receiving LAMB at 20 mg/kg once i.v. (24 h postinoculation). All data are means ± standard deviations of results from groups of three mice. The terminal half-life in the plasma and cerebrum is approximately 133 h.

A profound and durable antifungal effect was apparent following a single dose of 20 mg/kg in mice (Fig. 2). There was no evidence of significant fungal regrowth after 240 h of observation. The persistent antifungal effect may be explained by the long terminal half-life of amphotericin B in the plasma and cerebrum (∼113 h) (Fig. 3).

### Histopathology and immunohistochemistry in mice.

**FIG 4:**
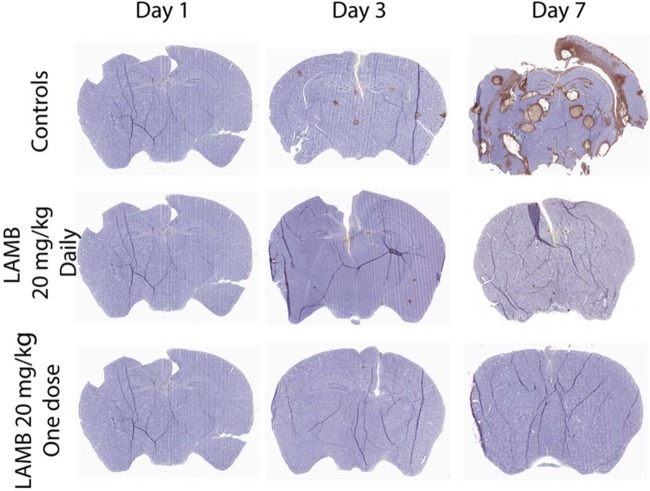
Representative cross-sections of brains from mice receiving vehicle control, LAMB at 20 mg/kg/day i.v., and LAMB at 20 mg/kg once i.v. Each section was stained with an anticryptococcal antibody and then counterstained with hematoxylin. Treatment was initiated at the end of day 1 (i.e., 24 h postinoculation). There are multiple cryptococcomas at the end of day 7 in untreated controls. In contrast, there are relatively few and exceedingly small lesions in the two treatment groups.

The persistent antifungal effect evident from the log_10_ CFU/g data were mirrored by the histopathological findings shown in Fig. 4. In mice receiving vehicle only, cryptococcal meningoencephalitis manifested as a multifocal disease with cyst-like cavities filled with multiple encapsulated organisms approximately 6 to 10 μm in diameter. There was no evidence of an inflammatory component within or around the cavities.

Mouse livers (harvested from mice receiving a total cumulative liposomal amphotericin B dose of 225 mg/kg) were used as the positive-control tissue in all amphotericin B localization experiments. Moderate to marked staining of frequent Kupffer cells was observed in the positive-control tissue. All other tissue elements were negative. There was no staining of Kupffer cells when a species-, isotype-, and concentration-matched negative-control antibody (rabbit IgG) was substituted for the rabbit anti-AMB reagent. Kuppfer cells in control mouse liver tissue that received 5% dextrose did not stain with rabbit anti-AMB reagent.

**FIG 5:**
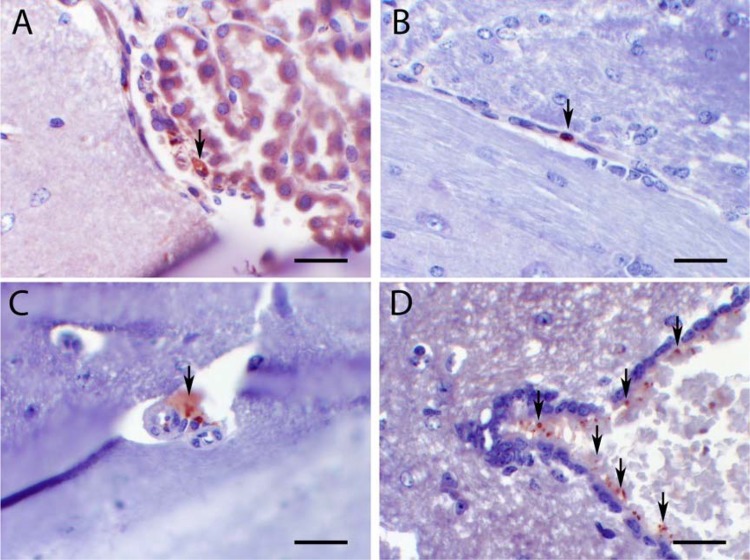
Distribution of LAMB in the central nervous system. All mice received LAMB at 20 mg/kg i.v. In each panel, black arrows indicate areas of staining of amphotericin B. (A) LAMB staining in the choroid plexus (extracellular and within a macrophage); (B) positive LAMB staining within a mononuclear cell in a thin-walled cerebral capillary; (C) positive staining in a perivascular location, adjacent to a thick-walled small arteriole; (D) LAMB staining in CSF associated with the apical surface of ependymal cells, cerebral aqueduct. Scale bars, 5 (A, B, and D) and 25 (C) μm.

There was differential penetration of amphotericin B into the brain (Fig. 5). Staining was apparent early (i.e., 1 h after dose) and in both intravascular and perivascular spaces, suggesting that the drug crossed the blood-brain barrier. Staining was especially prominent in blood vessels in the leptomeninges and choroid plexus, as well as small cerebral capillaries. Staining was both extra- and intracellular. Granular extracellular staining was observed in and surrounding blood vessels. Intracellular cytoplasmic staining was observed in mononuclear/microglial cells. Positive circulating mononuclear cells (presumptive monocytes) were identified in cerebral capillaries. Additional extracellular staining was observed in the ventricular system associated with the ependymal lining, suggesting entry into the CSF. In contrast, staining was not observed in the normal cerebral tissue or in residual cryptococcomas after 10 days of treatment with LAMB at doses of 10 or 20 mg/kg/day.

### Mathematical PK-PD model in mice.

The fit of the mathematical model to the combined murine pharmacokinetic-pharmacodynamic (PK-PD) data set was acceptable, even though fitting was difficult. The estimates for the parameters are summarized in Table 1. The principal challenge was modeling the depot-like effect of LAMB in the brains of mice in which low drug concentrations were observed to have exerted an antifungal effect that lasted well beyond the time that liposomal amphotericin B concentrations were detectable.

**TABLE 1:** Parameters from the population PK-PD model from mice^*a*^

Parameter (unit)	Value		
	Mean	Median	SD
CL_S_ (liters/h)	0.00082	0.00094	0.00018
Vol (liters)	0.003	0.0027	0.0019
*K*_cp_ (h^−1^)	11.99	10.52	9.94
*K*_pc_ (h^−1^)	15.70	23.33	11.41
*K*_cb_ (h^−1^)	0.16	0.22	0.013
*K*_bc_ (h^−1^)	0.034	0.01	0.038
*K*_g max_ (log_10_ CFU/g/h)	0.096	0.084	0.033
*H*_g_	7.96	7.72	6.00
*C*_50 g_ (mg/liter)	0.088	0.056	0.100
Pop_max_ (CFU/g)	23,785,100	57,435,030	249,007
*V*_m_ (liters)	0.72	0.94	0.37
Initial condition (CFU/g)	186	207	133.97

a Estimates for the mean, median, and standard deviation (SD) are shown. See the text for descriptions of parameters.

### PK-PD relationships in rabbits.

The mean parameter values best accounted for the observed PK data. The parameter values were as follows: clearance of drug from the central compartment (CL_S_), 0.018 ± 0.008 liters/h; first-order rate constant connecting the central and peripheral compartments (*K*_cp_), 10.37 ± 0.416 h^−1^; first-order rate constant connecting the peripheral and central compartments (*K*_pc_), 26.09 ± 0.96 h^−1^; first-order rate constant (*K*), 0.093 ± 0.04 h^−1^; initial volume (*V*_ini_), 4.717 ± 0.233 liters; and final volume (*V*_fin_), 0.003 ± 0.002 liters. The coefficients of determination for the linear regression before and after the Bayesian step were 0.87 and 0.98, respectively, and in both cases, the intercept and slope approximated zero and one, respectively.

**FIG 6:**
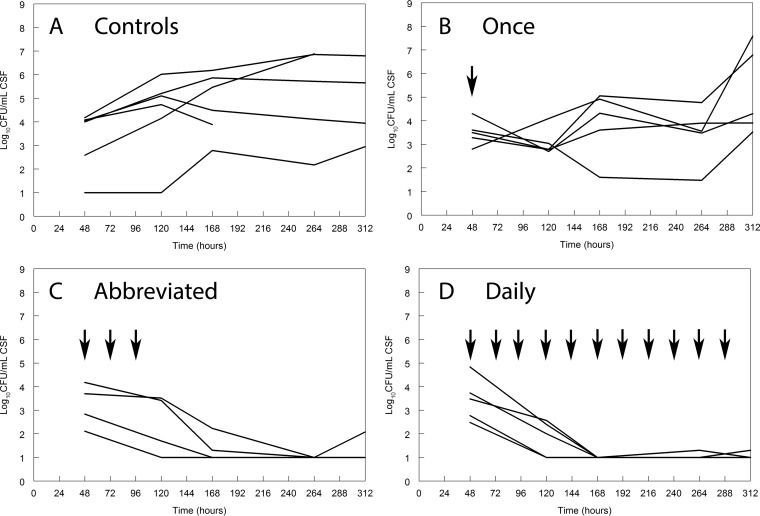
Time course of fungal density in the CSF of rabbits following various regimens of liposomal amphotericin B. Each line represents the data from a single rabbit. Each animal received 5 mg/kg every 24 h. The time course of fungal density in the CSF in rabbits receiving a single dose of drug (B) is comparable to that of controls (A). An abbreviated regimen of 5 mg/kg/day for three doses results in prompt fungicidal activity that is comparable to that achieved with daily therapy with the same dose.

The pharmacodynamics data in rabbits similarly illustrated the potential utility of abbreviated LAMB induction but differed somewhat from those observed in mice. A single dose of LAMB at 5 mg/kg appeared fungistatic only up to 264 h and did not provide a durable response in the CSF or cerebrum (Δlog_10_ CFU/g = 1.9 ± 1.2 and 3.2 ± 0.5, respectively), despite a higher estimated AUC from 0 to 24 h (AUC_0–24_) than that in mice receiving a single dose of 20 mg/kg (820 ± 15 versus 580 ± 30 mg · h/liter). Three doses of LAMB at 5 mg/kg administered every 24 h (and commencing 48 h postinoculation) induced a prompt decline in fungal burden in the CSF and cerebrum (Δlog_10_ CFU/ml = −2.8 ± 0.8 and −0.1 ± 0.4, respectively). This regimen produced a cumulative total AUC_48–120_ of 2,499 mg · h/liter. The effect of this abbreviated regimen in rabbits was comparable to that achieved with daily therapy (Fig. 6).

The exposure-response relationships in the cerebra of rabbits were similar. The fungal densities for control rabbits and for rabbits receiving 5 mg/kg once, 5 mg/kg/day for 3 days, and 5 mg/kg/day were 5.92 ± 0.55, 5.21 ± 1.20, 2.43 ± 1.23, and 2.47 ± 0.70 log_10_ CFU/g (mean ± standard deviation), respectively. Thus, in comparison to the murine studies, >1 day of therapy was required in rabbits to achieve fungicidal activity in the cerebrum and CSF.

### PK-PD targets and bridging studies.

A human regimen of liposomal amphotericin B of 4 mg/kg/day produces an AUC_0–24_ at steady state of ∼190 mg · h/liter. As can be seen in Fig. 1F, this is associated with near-maximal antifungal efficacy in mice receiving daily liposomal amphotericin B. A single dose of 20 mg/kg in mice (AUC_0–24_, 550 to 600 mg · h/liter) also produced near-maximal antifungal activity. The bridging study in rabbits suggested that a single dose of 5 mg/kg (AUC, 833 mg · h/liter) was insufficient to achieve fungicidal activity. Rather, a total of three doses of 5 mg/kg/day (cumulative AUC, 2,499 mg · h/liter) was required to achieve fungicidal activity in the CSF. Thus, there was a degree of discordance between the pharmacodynamic targets from mice and rabbits, with the latter requiring slightly more drug exposure to achieve the same effect.

## DISCUSSION

Amphotericin B is the most potent agent for induction therapy against Cryptococcus neoformans, and the combination with flucytosine results in the most rapid overall decline in fungal burden (17). This study suggests that abbreviated regimens of liposomal amphotericin B may be feasible. This is primarily a function of a favorable pharmacokinetic profile with long terminal elimination phases in both the plasma and brain (half-life [*t*_1/2_], 133 h).

The apparent discordance between plasma concentrations of liposomal amphotericin B and its persistent anticryptococcal activity in the central nervous system (CNS) of both mice and rabbits is of considerable interest, although the underlying mechanism driving this phenomenon is not entirely clear. One possibility is that there are a limited number of binding sites for amphotericin B in the CNS. Once occupied, amphotericin B does not readily disengage from its binding sites, leading to a pharmacologically active depot of drug. A relatively short course of liposomal amphotericin B (e.g., 1 to 3 doses) is all that is required to fully occupy these binding sites and result in persistent antifungal activity. Further doses are simply redundant and only serve to increase the probability of toxicity. The persistent occupation of receptors results in a sustained antifungal response for many days, even after plasma concentrations have declined to undetectable levels.

Exactly how liposomal amphotericin B traffics into the various clinically relevant effect sites is not clear. The drug penetrates into CNS subcompartments that are structurally normal and with histological evidence of inflammation (e.g., the ependyma shown in Fig. 5D). The immunohistochemistry studies suggest that the transfer from blood to the CNS occurs relatively quickly (i.e., in the first 24 h), but they do not enable estimates of the rate of transfer of drug. We did not observe high concentrations of drug within cryptococcomas, where the blood-brain barrier is likely significantly disrupted, even though amphotericin B was readily quantifiable in homogenates of cerebral tissue of mice. This is probably because the amphotericin B immunoassay is relatively insensitive. We did not see any evidence of the drug being carried into cryptococcomas by inflammatory cells (the dump truck phenomenon), as is described for macrolides (18), although there was a very limited inflammatory response in this model.

While the study provides the experimental foundation for the concept of using abbreviated induction regimens of liposomal amphotericin B for cryptococcal meningitis, there is some uncertainty about the best regimen(s) for humans. Taken in isolation, the rabbit studies suggest that more than a single dose is required (with a cumulative AUC of >833 mg · h/liter). The AUC associated with an i.v. dose of 5 mg/kg in a rabbit is higher than that observed following administration of 20 mg/kg i.v. to a mouse (833 versus 555 mg · h/liter, respectively), for which prolonged antifungal activity in the cerebrum was observed (Fig. 1). Thus, the mouse studies may underestimate the total (cumulative) drug exposure required for fungicidal activity in humans. Estimates of appropriate regimens are further complicated by some uncertainty in the PK of higher doses of liposomal amphotericin B in humans. We recently described much greater drug exposures (maximum concentration of drug in serum [*C*_max_] and AUC) after multiple doses in at least some children receiving up to 10 mg/kg of LAMB (19), although we did not observe this phenomenon with intermittent high doses in adults (20). Further detailed PK studies of higher doses of LAMB are warranted.

The current study has several limitations and assumptions. First, we did not examine whether immunological effectors may have had an antifungal effect in addition to that of LAMB and whether this may have contributed to persistent antifungal activity observed with single doses. There was no evidence from the histopathological studies of an inflammatory infiltrate in either mice or rabbits (the latter is not shown). We extensively investigated this possibility in a recent study that examined the effect of abbreviated regimens of DAMB for cryptococcal meningoencephalitis, in which there was no evidence of immune-mediated antifungal killing (13). Second, we made an explicit assumption that the trafficking (both the rate and extent) of drug from the bloodstream to the site of infection is the same in mice, rabbits, and humans. Such an assumption is central to PK-PD bridging studies for all drug-pathogen combinations. In the majority of cases, this assumption is reasonable, but there are isolated examples where it is not (21). Third, there remains a degree of uncertainty regarding the lowest dose and shortest possible course of LAMB that is likely to be effective for patients with cryptococcal meningoencephalitis. We did not design this study to specifically address this question, which would have required many more animals. Finally, we did not examine optimal combinations of antifungal agents when one or both agents is administered as a short course.

Given the overwhelming cost and feasibility advantages of abbreviated induction therapy based on one or few doses of LAMB, clinical trials are now required to further test these ideas. A two-stage adaptive open-label phase II/III randomized noninferiority trial comparing alternative short-course LAMB regimens is under way and will be reported in 2017 (trial registration number ISRCTN10248064). These clinical trials will provide information for new therapeutic options for this neglected infection.

## MATERIALS AND METHODS

### Strain and *in vitro* susceptibility testing.

Cryptococcus neoformans var. grubii (ATCC 208821 or H99) was the challenge organism for experiments in mice and rabbits. The MIC testing was performed using European Committee on Antimicrobial Susceptibility Testing (EUCAST) and Clinical and Laboratory Standards Institute (CLSI) methodology. MICs were determined in three independently conducted experiments.

### Laboratory animal models of cryptococcal meningoencephalitis.

All murine studies were performed under UK Home Office project license PPL 40/3630 and had received prior approval by the ethics committee at the University of Liverpool. Two models of cryptococcal meningitis that provide complementary information on the time course of cryptococcal meningoencephalitis, and the response to treatment with LAMB, were used. The murine model has the advantage of being highly reproducible. In this model, the fungal burden in the cerebrum is the primary readout and quantitative counts in the CSF cannot be obtained. In contrast, the rabbit model enables the time course of fungal burden in the CSF, which is a clinically relevant subcompartment within the central nervous system, to be determined. The fungal burden in other central nervous system subcompartments is also available (e.g., cerebrum, vitreous, meninges) but only at the time of sacrifice.

For the murine model, immunosuppression is not required because mice are inherently susceptible to disseminated cryptococcal infection. An inoculum of 3 × 10^8^ CFU in 0.25 ml phosphate-buffered saline (PBS) was injected i.v. via the lateral tail vein, which results in a highly reproducible encephalitis. Mortality occurs in the latter part of the second week of infection, meaning that early death does not confound any assessment of fungal burden in the initial 7 to 10 days of infection. The intended inoculum was confirmed by using quantitative counts after each experiment. The limit of detection for quantitative culture was 1.2 log_10_ CFU/g.

A rabbit model of cryptococcal meningoencephalitis that was originally developed and described by Perfect et al. (15) was used to study the impact of abbreviated regimens on the time course of fungal burden in the CSF. Briefly, C. neoformans inocula were grown in yeast extract-peptone-dextrose (YPD) broth to a final concentration of (3 ± 0.25) × 10^8^ CFU/ml. Inoculum concentrations were estimated by optical density and confirmed by quantitative culture. Anesthetized rabbits were infected via cisternal injection with an inoculum volume of 0.3 ml. Rabbits were anesthetized at day 2, 5, 7, and 11 postinoculation. CSF was obtained via cisternal puncture. A 1-ml sample was removed at each time point. All rabbits were sacrificed at the end of the experiment, which was 13 days postinoculation. A final CSF sample was obtained immediately after sacrifice. In addition, the fungal burden in the cerebrum at the end of the study period was determined as an additional endpoint. Representative samples of cerebrum were homogenized in 2 ml of PBS. Homogenate and CSF were then plated onto Sabouraud dextrose agar (SDA) containing chloramphenicol.

### PK-PD studies.

The PK-PD relationships in mice were determined over the course of multiple, independently conducted experiments. The time course of infection in the cerebrum was determined using a destructive design in which groups of CD-1 mice (*n* = 3 per group) were sacrificed at predefined intervals between 0 and 240 h postinoculation. Treatment was commenced 24 h postinoculation. Dose finding studies were conducted using 0.5 to 20 mg/kg/day. Each experiment incorporated an untreated control and at least two experimental arms (*n* = 15 per arm). Each dosing regimen was repeated in triplicate. Data from subjects requiring sacrifice on humane grounds were included in analyses at the time of death.

The PK data in plasma and cerebrum were determined in a separate experiment and once the relevant dose-response relationships had been determined. The PK was determined in infected mice. PK data were obtained at two intervals (immediately following the initiation of therapy and then after 5 days of dosing). Groups of mice (*n* = 3) were sacrificed 0.5, 1, 2, 6, and 24 h after drug administration. Plasma was obtained by terminal cardiac puncture, placed immediately on ice, centrifuged, and stored at −80°C for analysis. The cerebrum was extracted at the time of necropsy under sterile conditions. One hemisphere was submitted for quantitative cultures, while the other was stored for future measurement of amphotericin B concentrations.

PK-PD relationships were studied following various induction regimens, as follows. A single dose of 5 mg/kg was studied based on previous studies in invasive pulmonary aspergillosis. Groups of rabbits received a single dose, 3 doses of 5 mg/kg/day, and daily therapy of 5 mg/kg/day.

### Measurement of amphotericin B concentrations.

The concentrations of amphotericin B were estimated using a previously described assay (3). The limit of detection was 0.05 mg/liter. The intra- and interday variation was <7%.

### Histopathology and staining of LAMB in the CNS of mice.

The brain was collected and placed into 10% neutral buffered formalin for histopathologic evaluation. Formalin-fixed tissues were trimmed, cryoprotected by sucrose replacement, and then embedded in OCT freezing medium. Approximately 5-μm sections were prepared for staining.

A commercially available mouse monoclonal antibody directed against Cryptococcus neoformans was used to determine the extent of infection (MyBioSource, LLC, San Diego, CA). Yeasts stained consistently and intensely positive with the anti-C. neoformans antibody. The yeasts did not stain with the species-, isotype-, and concentration-matched negative-control antibody (mouse IgG1) that was substituted for the mouse anti-C. neoformans reagent (data not shown).

Amphotericin B was visualized using an affinity-purified rabbit anti-amphotericin B antibody (Antibodies Inc., Davis, CA). Immunohistochemistry was performed using standard immunoperoxidase and alkaline phosphatase methodology and validated by appropriate and reproducible positive and negative controls for staining amphotericin B as previously described (22, 23).

### Mathematical modeling.

The murine PK and PD data from mice were modeled using a population methodology with the program Pmetrics (24). The mean drug concentration, cerebral concentration, and fungal burden in the cerebra from groups of three mice were used. All data were weighted by the observed variance from each group of mice for drug concentrations and fungal burden. The structural model took the form:

(1) $$\frac{{dX}_{1}}{dt}=R_{1}-\left(\frac{{\text{CL}}_{\text{S}}}{V_{\text{C}}}+K_{\text{cp}}+K_{\text{cb}}\right)\times X_{1}+K_{\text{bc}}\times X_{3}+K_{\text{pc}}\times X_{2}$$

(2) $$\frac{{dX}_{2}}{dt}={-K}_{\text{pc}}\times X_{2}+K_{\text{cp}}\times X_{1}$$

(3) $$\frac{{dX}_{3}}{dt}=K_{\text{cb}}\times X_{1}-K_{\text{bc}}\times X_{3}$$

(4) $$\frac{dN}{dt}=K_{\text{g }\max}\times (1-\left(\frac{\frac{{X_{3}}^{H_{\text{g}}}}{V_{\text{m}}}}{\frac{{X_{3}}^{H_{\text{g}}}}{V_{\text{m}}}+{C_{50\text{ g}}}^{H_{\text{g}}}}\right))\;\times \;\left(1-\frac{N}{{\text{Pop}}_{\max}}\right)\times N$$

where *X*_1_, *X*_2_, and *X*_3_ represent the amounts of amphotericin B (in milligrams) in the central compartment, peripheral compartment, and cerebrum, respectively. *N* is the number of organisms in the cerebrum. *R*_1_ represents the i.v. injection of liposomal amphotericin B in milligrams; *K*_cb_, *K*_bc_, *K*_cp_, and *K*_pc_ represent the first-order rate constants connecting the central (c), peripheral (p), and cerebrum (brain [b]) compartments. *H*_g_ is the slope function for the suppression of growth. *K*_g max_ is the maximum rate of fungal growth in the brain, *V*_m_ is the volume of the murine brain, *V*_c_ is the volume of the central compartment, *C*_50 g_ is the concentration of amphotericin B in the brain at which there is half-maximal inhibition of growth, and Pop_max_ is the maximum theoretical density of organisms in the brain.

Equation 1 describes the movement of liposomal amphotericin B into and out of the central compartment (plasma). Equation 2 describes the movement of liposomal amphotericin B into and out of the peripheral compartment. Equation 3 describes the movement of drug into the brain. Equation 4 describes the pharmacodynamics of amphotericin B. This equation contains terms that describe the capacity-limited fungal growth in the brain and drug-induced suppression of the fungal growth.

The fit of the mathematical model to the combined PK and PD data set from mice was assessed using the log likelihood value, measures of precision and bias, visual inspection of the observed-versus-predicted values both before and after the Bayesian step, and assessment of the linear regression of the observer-versus-predicted values both before and after the Bayesian step. Inspection of the PK data of liposomal amphotericin B in rabbits suggested that the volume of distribution contracted with time, as recently described by us in children (19).

(5) $$\frac{{dX}_{1}}{dt}=R_{1}-\left(\frac{{\text{CL}}_{\text{S}}}{X_{3}}+K_{\text{cp}}\right)\times X_{1}+K_{\text{pc}}\times X_{2}$$

(6) $$\frac{{dX}_{2}}{dt}=K_{\text{cp}}\times X_{1}-K_{\text{pc}}\times X_{2}$$

(7) $$\frac{{dX}_{3}}{dt}=-X_{3}\times K+V_{\text{fin}}$$

with output equation

(8) $$Y_{1}=X_{1}/X_{3}$$

where *X*_1_ and *X*_2_ are the amounts of liposomal amphotericin B in the central and peripheral compartments, respectively. CL_S_ is the clearance of drug from the central compartment, and *K*_cp_ and *K*_pc_ are the two first-order intercompartmental rate constants connecting the central (c) and peripheral (p) compartments. *X*_3_ is the volume of the central compartment that contracts with time according to equation 7. *X*_3_ has an initial volume, *V*_ini_, which is estimated as an initial condition in Pmetrics. The volume contracts over time according to the first-order rate constant, *K*. The final volume after prolonged drug administration is *V*_fin_. *Y*_1_ is the concentration of liposomal amphotericin B in the central compartment. Equation 5 describes the rate of change of the amount of liposomal amphotericin B in the central compartment. Equation 6 describes the rate of change of the amount of liposomal amphotericin B in the peripheral compartment.

### PK-PD bridging studies.

In order to place the experimental findings in a clinical context, we bridged the preclinical PK-PD findings from mice and rabbits to patients using a previously described population PK model for liposomal amphotericin B (20). This model was used to estimate the average drug exposure (quantified in terms of AUC) resulting from various human doses.
